# Outdoor Exercise Facility–Based Integrative Mobile Health Intervention to Support Physical Activity, Mental Well-Being, and Exercise Self-Efficacy Among Older Adults With Prefrailty and Frailty in Hong Kong: Pilot Feasibility Randomized Controlled Trial Study

**DOI:** 10.2196/69259

**Published:** 2025-06-05

**Authors:** Janet Lok Chun Lee, Arnold Y L Wong, Peter H F Ng, S N Fu, Kenneth N K Fong, Andy S K Cheng, Karen Nga Kwan Lee, Rui Sun, Hao Yi Zhang, Rong Xiao

**Affiliations:** 1 Department of Rehabilitation Sciences The Hong Kong Polytechnic University Hong Kong China (Hong Kong); 2 Research Institute for Smart Ageing The Hong Kong Polytechnic University Hong Kong China (Hong Kong); 3 Mental Health Research Centre The Hong Kong Polytechnic University Hong Kong China (Hong Kong); 4 Research Institute for Sports Science and Technology The Hong Kong Polytechnic University Hong Kong China (Hong Kong); 5 Research Centre for Assistive Technology The Hong Kong Polytechnic University Hong Kong China (Hong Kong); 6 Department of Computing The Hong Kong Polytechnic University Hong Kong China (Hong Kong); 7 School of Health Sciences Western Sydney University Sydney Australia; 8 Jockey Club Design Institute for Social Innovation The Hong Kong Polytechnic University Hong Kong China (Hong Kong)

**Keywords:** integrative intervention, mHealth, outdoor exercise facilities, physical activity, built environment, frailty, community-dwelling older adults

## Abstract

**Background:**

Engaging in an adequate amount of physical activity (PA) serves as a protective factor against frailty. While previous PA interventions have been effective in improving physical functioning outcomes, they have not consistently succeeded in sustaining PA behavioral changes.

**Objective:**

The primary aim of this pilot randomized controlled trial (RCT) is to explore the feasibility and acceptability of an integrative mobile health (mHealth) intervention among community-dwelling older adults with prefrailty and frailty. The secondary aim was to investigate the potential effects of the intervention on sustaining PA levels and improving mental well-being and exercise self-efficacy in this population.

**Methods:**

A 2-armed pilot feasibility randomized controlled trial was conducted. A total of 38 inactive, community-dwelling older adults (aged>55 years) with prefrailty and frailty were randomized to either the intervention group (n=19), which received 4 weekly educational workshops at a university and a mobile app to support their use of outdoor exercise facilities in their neighborhood, or the control group (n=19), which received 4 weekly health education workshops with exercise experiential sessions tailored for older adults with frailty. To assess the acceptability of the intervention, individual semistructured interviews were conducted with, and a self-developed questionnaire was administered to, 14 participants from the intervention group.

**Results:**

The mean age of the participants was 71.8 (SD 9.34) years, and 24 out of 34 (71%) were female. As many as 34 participants out of 38 (89%) completed the study (18/19 in the control group and 16/19 in the intervention group). Workshop attendance rates were very high in both groups (intervention group, 63/68, 93%, and control group, 72/76, 95%). Self-reported adherence to the unsupervised outdoor practical sessions and engagement with the app was over 65% (36/51, 71%, and 35/51, 69%, in the intervention group. Two adverse events were reported in the intervention group, and none in the control group. As hypothesized, secondary outcome analyses showed that both groups increased their PA levels immediately after the intervention; however, only the intervention group maintained this increase at the 3-month follow-up. Additionally, favorable changes in mental well-being and exercise self-efficacy were observed in the intervention group. Feasibility and acceptability data also highlighted areas for improvement that should be addressed before a larger trial.

**Conclusions:**

This study provides initial proof-of-concept evidence for the integrative mHealth intervention. However, modifications are needed to enhance user adherence to both the mobile app and the outdoor practice component before proceeding to a larger trial.

**Trial Registration:**

ClinicalTrials.gov NCT06326710; https://clinicaltrials.gov/ct2/show/NCT06326710

## Introduction

### Background

Population aging is a global phenomenon and an irreversible trend. Statistics show that by 2050, the world population of individuals aged 65 years or older will surpass 1.6 billion [1]. It is also estimated that 20% of the aged population in Asia [2], and 16.6% of older adults in Hong Kong, are frail [3]. Frailty affects various aspects of older adults’ lives, including gait, mobility, balance, muscle strength, motor processing, cognition, nutrition, endurance, and physical activity (PA) [4]. Importantly, high levels of frailty increase the risks of adverse health outcomes such as falls, hospitalization, and mortality [5].

Frailty is a modifiable, dynamic process characterized by frequent transitions between different frailty states over time. Research has shown that interventions and health strategies can be effectively used to prevent, delay, or even reverse frailty among community-dwelling older adults [6]. PA interventions are considered one of the main strategies to counteract frailty-related physical impairments in older adults [7]. Engaging in an adequate level of PA has been identified as a protective factor against frailty in this population [8]. Participating in regular PA improves the quality of life of older adults with frailty by enhancing balance and mobility, and by reducing the risks of falls, hospitalization, institutionalization, and mortality [9]. Therefore, it is crucial to develop effective and sustainable PA interventions for community-dwelling older adults with frailty.

According to review studies, most PA interventions designed for older adults with frailty have demonstrated effectiveness in improving functional performance and reducing the incidence of falls. However, they have not consistently succeeded in facilitating sustainable behavioral changes related to PA [10,11]. For instance, a 14-week exercise intervention that used wearable activity trackers and incorporated behavioral change techniques for older adults with frailty succeeded in improving physical performance 3 months after the intervention but did not enhance PA levels [12].

In recent years, outdoor exercise facilities (OEFs) have become increasingly prevalent worldwide as a means of promoting PA among citizens. Interventions have also been developed to educate older adults on using OEFs to improve health outcomes such as muscle strength, balance, mobility, PA levels, and weight control [13,14]. OEF-based interventions have demonstrated effectiveness in supporting sustained behavioral changes related to PA [13,15]. More recently, an OEF-based intervention integrated with mobile health (mHealth) was found to be effective in promoting and sustaining resistance training–specific PA changes in adults [16].

Since 2002, the Hong Kong government has been installing senior-friendly OEFs throughout the territory. Unlike in other regions, where OEFs have been designed and installed for the general adult population, those in Hong Kong have been specifically tailored to meet the needs of older adults. The exercise equipment is low impact and nonresistance, aiming to provide a safe and accessible environment for seniors [17]. Statistics show that senior-friendly OEFs have now been installed at more than 440 locations across Hong Kong [18]. Because of the nonresistance nature of the equipment, exercising with these OEFs has been objectively shown to be of low-to-moderate intensity [19]. Additionally, an in-depth qualitative investigation revealed that older adults with frailty in Hong Kong preferred using OEFs, as they perceived the exercise intensity to be “nonvigorous” [20].

Despite the availability of numerous senior-friendly OEFs in Hong Kong, older adults—especially those with frailty—may hesitate to use or may misuse these facilities due to a lack of proper training. To address this limitation, an integrative mHealth intervention can be implemented to support the proper use of OEFs by community-dwelling older adults with frailty in Hong Kong and to foster sustainable behavioral changes related to PA. Research has shown that integrative mHealth interventions can facilitate the development of sustainable PA-related behavioral changes in the general population [21]. Recent studies also indicate that older adults hold a positive perception of mHealth, provided they receive social support and instructional guidance [22]. The proposed integrative mHealth intervention incorporates educational workshops and supports unsupervised outdoor self-practice sessions in participants’ neighborhoods via a mobile app, demonstrating its potential to enhance and sustain PA levels, as well as to improve mental well-being and exercise self-efficacy among inactive and community-dwelling older adults with frailty. The primary aim of this pilot randomized controlled trial (RCT) was to explore the feasibility and acceptability of the integrative mHealth intervention among community-dwelling older adults with prefrailty and frailty. The secondary aim was to examine the potential effects of the intervention in sustaining PA levels and improving mental well-being and exercise self-efficacy in this population. It was hypothesized that both groups would show improvement in PA immediately after the intervention, but only the intervention group would maintain increased PA levels over time. Additionally, it was hypothesized that the intervention group would demonstrate greater improvements in mental well-being and exercise self-efficacy compared with the control group.

## Methods

### Trial Design

This study was reported in accordance with the CONSORT (Consolidated Standards of Reporting Trials) statement extension for pilot trials [23]. Please refer to Multimedia Appendix 1 for the CONSORT checklist. This study was a prospective, 2-armed, single-blinded (participant-blinded), parallel-group pilot RCT with an allocation ratio of 1:1. A qualitative interview was conducted with participants in the intervention group following the pilot RCT. The trial was conducted between May and September 2024 at a university in Hong Kong and was registered on ClinicalTrials.gov (March 22, 2024).

### Participants

Participants were recruited in April 2024 using convenience sampling. Recruitment was conducted through public talks delivered online, at community health centers, and at health promotional booths. Enrollment screening was carried out by the research assistant according to the inclusion and exclusion criteria (Textbox 1).

**Inclusion and exclusion criteria for participants.**

**1. Inclusion criteria**

- Community-dwelling older adults aged 55 years or over.
- Self-report of frailty or prefrailty as determined by a score of ≥1 and ≤5 on the Chinese version of the FRAIL (Fatigue, Resistance, Ambulation, Illness, and Loss of Weight) scale [24].
- A score of 0 out of 7 on the Physical Activity Readiness Questionnaire [25].
- Use a smartphone on a daily basis.

**2. Exclusion criteria**

- Those who have already achieved an adequate level of daily physical activity, defined as engaging in at least 150 minutes of moderate-to-vigorous physical activity per week by a single-item question with a dichotomous response (ie, yes/no). They were also asked to provide their exercise habits to validate their response to this single-item question.

### Sample Size

This was a pilot feasibility trial; therefore, it was not designed to test the effect of the intervention, which requires a fully powered study. Instead, this pilot trial aimed to estimate the potential effects in a small sample, providing a reference for calculating the sample size of the main trial.

The determination of the sample size for this study was based on 2 key factors: Hertzog’s sample size recommendation [26] and insights from recent mHealth studies using a pilot RCT design. Hertzog suggested that pilot studies should include between 10 and 40 participants per group [26]. While this range is broad, the research team also considered recent mHealth studies focusing on PA behavioral change to guide the decision on sample size. Recent mHealth studies with a similar focus on PA behavior reported that recruiting and randomly allocating between 33 and 42 participants to the intervention and control groups was sufficient to assess preliminary differences between groups [27,28]. Considering these factors, this study aimed to recruit a total of 30-40 participants for random allocation. It is important to note that this study was not powered to detect significant changes in secondary outcomes; instead, the mean difference between groups observed at the 3-month follow-up, along with the SD, will be used to estimate the sample size for a future large-scale definitive RCT.

### Intervention

The integrative mHealth intervention group comprised 3 components: (1) a mobile app [29], (2) educational workshops, and (3) unsupervised outdoor self-practice sessions. Participants in the intervention group attended 4 weekly 1-hour educational workshops led by an interventionist with expertise in exercise and health. These workshops covered various topics, including how to use the app to identify OEFs in their neighborhood and how to correctly use each OEF. Additional workshop content addressed risk management during outdoor exercise, the association between frailty and exercise, selecting appropriate exercise intensity, strengthening and balance exercises, postural awareness, pain management during exercise, and principles for modifying exercises when using OEFs. Over the 4-week intervention period, participants were encouraged to use the OEFs with the assistance of the app to learn the correct usage of the facilities. During the workshops, they had the opportunity to share their experiences using the app and discuss any challenges encountered during the unsupervised outdoor practice sessions. Their adherence to the outdoor practice sessions was recorded by the research assistant at each workshop.

### The Outdoor Rehab-Fit App

The Outdoor Rehab-Fit App was developed by PA researchers, clinician-scientists, and computer scientists at the Department of Rehabilitation Sciences, The Hong Kong Polytechnic University. The app offers (1) an OEF library, (2) OEF locations, and (3) risk management guidance for outdoor exercise, among other features. For each piece of outdoor exercise equipment in the library, the app provides the health benefits of using the equipment, tips from a physiotherapist and an occupational therapist, and advice for caregivers—all presented in text format. This additional health information, tailored to each piece of equipment by exercise and health professionals, aims to enhance users’ perceived benefits and outcome expectancies of using the equipment for PA. Additionally, an audio guide provides step-by-step voice-over instructions to help users learn about the OEFs. Short videos for each piece of equipment are also available to remind users of the correct postural alignment while exercising. These multimedia formats improve users’ self-efficacy in performing the exercises.

### Active Control

Participants in the active control group attended four 60-minute health education workshops led by an interventionist with expertise in exercise and physical health management. These workshops focused on the benefits of various exercises for older adults, particularly those who are frail. In addition to health knowledge, participants were given opportunities to try different modified exercises suitable for older adults with frailty, such as chair yoga, seated Baduanjin, Otago exercises, and seated fitness exercises. The structure and content of these workshops mirrored common community-based programs promoting PA in older adults. This comparator was selected to compare the integrative mHealth intervention against existing practices. Additionally, by providing structured sessions with the same exercise theme to both groups, the comparator minimized potential confounding factors such as participant expectations. Finally, the active control ensured ethical fairness by offering all participants support for PA.

### Outcomes

#### Primary Outcomes

##### Overview

The primary outcomes were the feasibility, safety, and acceptability of the intervention.

##### Feasibility

The feasibility of the integrative mHealth intervention was assessed based on the recruitment rate, intervention completion rate, and attrition rate. The recruitment rate refers to the percentage of individuals who signed up for the intervention out of all those who were approached. The intervention completion rate reflects the extent to which participants completed all 3 components (app, workshops, and outdoor self-practice) in the intervention group, or attended the education talks in the control group, during the 4-week intervention period. Intervention completion was recorded using a self-developed form and calculated as the percentage of intervention components completed (Multimedia Appendix 2).

##### Safety

Any adverse events occurring in either group during the intervention and follow-up periods were recorded. These events included incidents such as falls and muscle or joint pain, as well as serious occurrences such as difficulty breathing, new or persistent chest pain, or sudden changes in consciousness that require immediate medical attention.

##### Acceptability

The acceptability of the app and the integrative mHealth intervention were evaluated using a self-developed questionnaire (Multimedia Appendix 3) and a semistructured interview. The interview guide was developed based on a similar mHealth intervention study [30] (Multimedia Appendix 4). The interviews gathered participants’ overall views of the intervention, perceived barriers and facilitators, and recommendations, and were conducted either face-to-face or online.

#### Secondary Outcomes

##### Overview

The secondary outcomes included measures of PA behaviors (utilization of OEFs and overall PA), exercise self-efficacy, and mental well-being. These measures were selected to assess the intervention’s effectiveness across multiple domains.

##### PA Behaviors

- Participants’ utilization of OEFs was assessed using a self-developed questionnaire that collected information on the frequency, duration, and types of OEFs typically used.
- The Rapid Assessment of Physical Activity Scale (RAPA) [31] was used to measure self-reported PA. RAPA is commonly used in community-based interventional studies [32,33]. It consists of 9 yes-or-no items and evaluates PA levels ranging from sedentary to regular vigorous activity, as well as exercise types. The questionnaire has 2 sections: RAPA_1_ and RAPA_2_. RAPA_1_ includes 7 questions related to the level of leisure-time aerobic activity, illustrated with pictures depicting light, moderate, and vigorous activities. RAPA_2_ consists of 2 questions focusing on strength and flexibility training. RAPA has 81% sensitivity, 69% specificity, and a 77% positive predictive value. It is significantly (*P*<.001) correlated with the frequently used Community Healthy Activities Model Program for Seniors Physical Activity Questionnaire (CHAMPS) for older adults. The PA level assessed by RAPA_1_ can be treated as a continuous variable to observe changes over time [33]. This study used only the data from RAPA_1_ to examine changes in aerobic activity over time. RAPA was chosen because it requires less time to administer and imposes a lower cognitive load compared with CHAMPS. Given that the participants are older adults with frailty, RAPA was selected to minimize their burden and reduce respondent fatigue.
- Objective measurement of moderate-to-vigorous intensity physical activity (MVPA) was collected using an accelerometer (ActiGraph GT3X, ActiGraph LLC). This activity monitor has been validated and shown to have good criterion validity against calorimetry in distinguishing MVPA from non-MVPA during treadmill walking at various speeds [34]. The monitor was worn on the participant’s nondominant wrist for 7 consecutive days during waking hours, with a minimum of 10 hours of wear time each day [35,36]. A 7-day monitoring period is recommended to capture typical daily PA patterns [35,37]. Literature indicates that wrist-worn activity monitors outperform hip-worn monitors when participants walk at speeds of 3-6 km/h [34]. Participants received instructions on how to properly wear the monitor on their nondominant wrist before the assessment. Participants were allowed to remove the ActiGraph during specific activities such as bathing, swimming, and sleeping. They also maintained a daily wear-time log to record the monitor’s on and off times throughout the monitoring period. Participants were instructed not to engage in any additional PA beyond their usual routine while wearing the monitor. A research assistant contacted participants on the first or second day after device distribution to ensure proper use [38]. The ActiGraph devices were initialized to collect raw data at a sampling rate of 60 Hz. After screening for data validity, only complete and valid data sets were processed using ActiLife software in low-frequency extension mode [39-41]. Valid data were defined as wear time exceeding 10 hours per day for at least 5 days, or totaling more than 50 hours across 4 days [35]. Activity counts per minute from the monitor were converted into minutes of MVPA using age-specific cutoff points established in the literature, specifically 4117.7 counts or more per minute [34]. These cutoffs were derived from a previous study [34] validating ActiGraph use in identifying PA intensity levels among the Hong Kong older adult population.
- The Chinese version of the Exercise Self-Efficacy Scale [42] was used to assess participants’ confidence in maintaining regular exercise. This scale comprises 9 items, each rated on an 11-point Likert scale from 0 (not confident) to 10 (very confident). The mean score of these items, ranging from 0 to 10, represents the overall exercise self-efficacy, with higher scores indicating greater confidence in regularly engaging in exercise.
- The mental well-being of participants was assessed using the Chinese version [43] of the 7-item Short Warwick-Edinburgh Mental Well-Being Scale (SWEMWBS) [44]. This scale addresses both hedonic and eudaimonic aspects of well-being, with items rated on a 5-point Likert scale from 1 (none of the time) to 5 (all the time). The mean score, calculated by averaging responses across the 7 items, reflects the overall mental well-being, with higher scores indicating greater well-being.

### Randomization and Blinding

Randomization was performed after the completion of the screening. Participants were randomly allocated to either the intervention or active control group on a 1:1 basis using block randomization by a research assistant. The randomization process did not consider whether participants were couples. Block sizes of 4 or 6 were randomly generated using random number generator software [45].

### Blinding

Participants were blinded to their group allocation and informed only that they had been assigned to 1 of 2 groups to evaluate the feasibility and effects of the exercise programs. All assessments were conducted by trained assessors with health sciences backgrounds.

### Statistical Analysis

#### Quantitative Data

Descriptive analyses were conducted to summarize demographic characteristics, frailty levels, chronic disease diagnoses, hospitalizations, history of falls in the past 12 months, and use of outdoor walking aids. Means and SDs were used to report continuous parametric data, while medians and IQRs summarized continuous nonparametric data. Categorical data were presented as frequencies and percentages. Recruitment rate, intervention completion rate, attrition rate, and attendance rate were reported as percentages. Adverse events were recorded and described narratively.

Although this study was not specifically powered to detect statistically significant differences between groups for the secondary outcomes, analyses were conducted to explore these differences. This approach aimed to inform the selection of outcome measures for a future fully powered clinical trial and to facilitate preliminary exploration of potential intervention effects. Data analyses followed the intention-to-treat principle. Descriptive statistics were used to examine statistical trends. Generalized estimating equations were used to analyze temporal changes and between-group differences in self-reported and objectively measured PA levels, exercise self-efficacy scores, and mental well-being scores. To account for multiplicity in pairwise comparisons of secondary outcomes, least squares difference correction was applied. Effect sizes were estimated using Cohen *d*, adjusted for generalized estimating equations analysis, based on the following formula: β_interaction_/SD_residual_.

#### Qualitative Data

Interviews were audio-recorded and transcribed verbatim. Thematic analysis was conducted following the 6-phase framework outlined by Braun and Clarke [46]. Data were inductively coded and then deductively organized into 4 key domains: (1) overall perception of the intervention, (2) facilitators, (3) barriers, and (4) suggestions for improvement. NVivo version 14 (QSR International Pty Ltd) was used to support data management and analysis. To enhance the credibility of the findings, analyst triangulation was applied. The first author conducted the initial coding and analysis, while all coauthors reviewed and refined the themes. Any discrepancies were resolved through discussion and consensus.

### Ethics Considerations

Ethical approval has been obtained from The Hong Kong Polytechnic University Institutional Review Board (PolyU IRB; reference number HSEARS20230306001-3). All participants will be required to sign a consent form before participation. Where a participant lacks the capacity to provide consent, a nominated representative will sign the consent form, along with obtaining assent from the participant.

Any information obtained in this study will remain strictly confidential and be used for research purposes only. Codes, not names, are used on all reports and publications related to this study to protect confidentiality. All collected questionnaires will be kept in locked cabinets. The electronic data set will be stored in encrypted computer storage. Data containing personal identifiers will be kept for a maximum of 3 years after publication of the first paper upon the end of the study.

### Dissemination Policy

Manuscripts resulting from this trial will be published in academic journals or abstracts of papers will be presented in academic conferences. Authorship eligibility includes (1) substantial contributions to the study design or the process of data acquisition, analysis, or interpretation, (2) drafting or revising the manuscript, and (3) approving the final manuscript. There is no intention to use professional writers.

## Results

### Demographic and Other Characteristics of the Participants

The demographic and health characteristics of the participants are presented in Table 1. No significant differences (*P*<.05 in all cases; see Table 1 for the complete *P* values) were observed between groups at baseline, except for the incidence of falls in the past 6 months (*P*=.04). Additionally, due to attrition occurring immediately after group allocation, there was an imbalance in gender distribution between the groups. To enhance the robustness of the results, both gender and incidence of falls were included as covariates in the statistical analyses.

**Table 1:** Demographic characteristics of the participants.

Characteristics			Total, n (%)		Intervention (n=16), n (%)		Control (n=18), n (%)		*P* value
**Age**									.09
	Mean (SD)	71.8 (9.34)		69.0 (8.18)		74.28 (9.82)			
	Median	70		66.5		72.5			
**Gender**									.20
	Male	10 (29)		3 (19)		7 (39)			
	Female	24 (71)		13 (81)		11 (61)			
**Education**									.38
	No formal education	2 (6)		0 (0)		2 (11)			
	Primary level	5 (15)		1 (6)		4 (22)			
	Secondary level	15 (44)		10 (63)		5 (28)			
	Tertiary level	8 (24)		2 (13)		6 (33)			
	Postgraduate level	4 (12)		3 (19)		1 (6)			
**Living arrangement**									.93
	Living alone	11 (32)		5 (31)		6 (33)			
	Living with family	23 (68)		11 (69)		12 (67)			
**Weight (kg)**									.75
	Mean (SD)	58.4 (9.36)		59.0 (8.19)		57.9 (10.5)			
	Median	59.5		59.5		59.5			
**Height (cm)**									.35
	Mean (SD)	155.4 (7.14)		157.6 (7.56)		155.2 (6.77)			
	Median	155.5		155.0		155.5			
**BMI (kg/m^2^)**									.82
	Mean (SD)	23.8 (2.48)		23.7 (2.04)		23.9 (2.87)			
	Median	23.1		23.0		23.6			
**Frailty level (FRAIL^a^ scale)**									.75
	Score=1	25 (74)		12 (75)		13 (72)			
	Score=2	4 (12)		3 (19)		1 (6)			
	Score=3	4 (12)		1 (6)		3 (17)			
	Score=4	1 (3)		0 (0)		1 (6)			
**Chronic disease**									.82
	Yes	24 (71)		11 (69)		13 (72)			
	No	10 (29)		5 (31)		5 (28)			
**Use walking aids outdoors**									.48
	Wheelchair	2 (6)		1 (6)		1 (6)			
	Walking stick	5 (15)		1 (6)		4 (22)			
	None	28 (82)		14 (88)		13 (72)			
**Hospitalized in the past 12 months**									1.00
	Yes	0 (0.0)		0 (0.0)		0 (0)			
	No	34 (100)		16 (100)		18 (100)			
**Fallen in the past 6 months**									.04
	Yes	4 (12)		0 (0)		4 (22)			
	No	30 (88)		16 (100)		14 (78)			

^a^FRAIL: Fatigue, Resistance, Ambulation, Illness, and Loss of Weight.

### Feasibility of the Intervention

**Figure 1:**
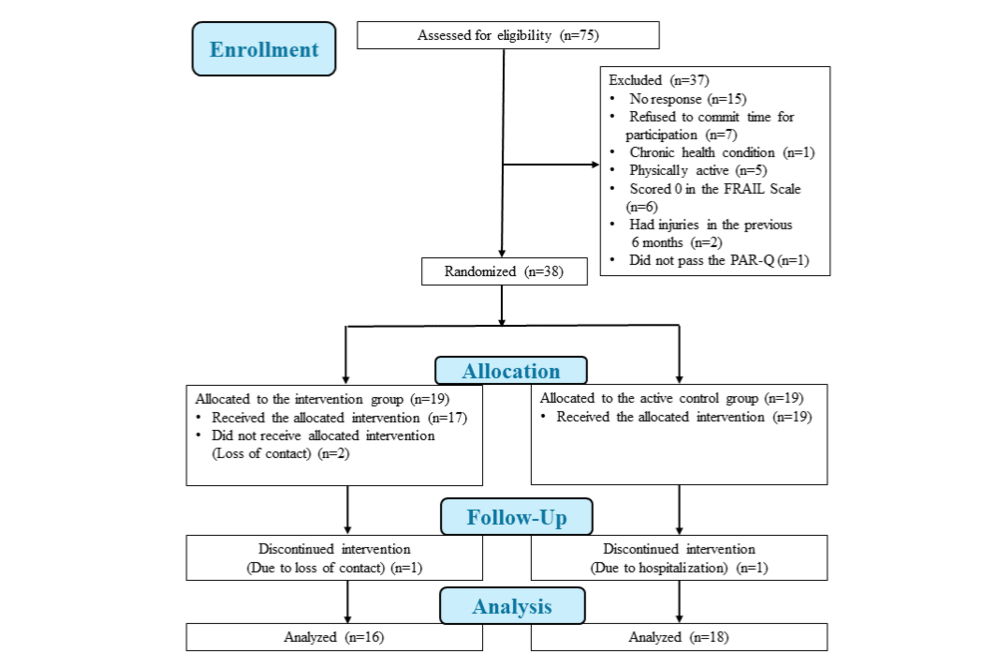
CONSORT (Consolidated Standards of Reporting Trials) flowchart.

Of the 75 individuals, 38 (51%) were recruited. Of the 75 individuals who met the eligibility criteria, 38 were randomized into the study groups. In the intervention group, 17 of the 19 participants received the integrative mHealth intervention, while 2 participants were lost to follow-up before the intervention began. All 19 participants allocated to the active control group received the control sessions. The attrition rate was less than 20% (3/19, 16%) in the intervention group and under 10% (1/19, 5%) in the active control group. Among the participants who dropped out, the reasons included loss of contact (n=3) and hospitalization (n=1; see Figure 1). The overall intervention completion rate in the integrative mHealth intervention group was nearly 80% (134/170, 78.8%). The average completion rates for individual components were as follows: workshop attendance, 63 out of 68 (93%); self-reported adherence to unsupervised outdoor practice sessions, 36 out of 51 (71%); and self-reported mobile app engagement, 35 out of 51 (69%). The active control group, which included only the workshop component, had a completion rate of over 90% (72/76, 95%). Two adverse events were reported in the intervention group, while none was reported in the control group. In the intervention group, 1 participant experienced an upper body slipping incident while using faulty exercise equipment. Another participant reported knee and lower back pain after using the stepper and twister; the discomfort was alleviated by modifying the exercise duration and reducing the range and speed of movement. No other adverse events related to the interventions were reported by participants.

### Acceptability of the App and the Integrative mHealth Intervention

A total of 14 participants from the intervention group, including 11 women and 3 men aged 61-77 years, with frailty levels ranging from 1 to 3, took part in the semistructured interviews. Additionally, 13 of these participants completed the self-developed questionnaire on the perceived usefulness of the app. The questionnaire results showed that all respondents agreed or strongly agreed that the app improved their exercise performance, and at least 12 (86%) of them agreed or strongly agreed that they intended to continue using the app to support their outdoor workouts. All respondents found the app’s “Introduction by an Exercise Specialist,” “Tips from a Physiotherapist,” “Tips from an Occupational Therapist,” and “My Favorites” features useful or extremely useful (Tables 2 and 3). Themes and subthemes related to participants’ overall perceptions of the intervention, as well as the barriers, facilitators, and suggestions for future improvements, were identified through qualitative analysis (Table 4). Participants appreciated the structured design of the intervention, which included the app, workshops, and follow-ups. They reported increased motivation and confidence in using the free-of-charge OEFs to self-manage their physical health. The intervention also enhanced their awareness of the health benefits associated with OEF use. Barriers and facilitators related to the various components of the integrative mHealth intervention—the app, outdoor practice, and workshops—were identified. For the app component, participants reported barriers such as small image sizes, the inability to geolocate certain outdoor exercise equipment, and the unavailability of some equipment listed in the app. Barriers to the outdoor practice component included equipment being out of order, hygiene concerns, lack of storage for personal belongings, disruptive behavior from other users, unfavorable weather conditions, and social factors. By contrast, the app’s content and the workshop’s delivery mode were viewed as key facilitators of engagement. To enhance the intervention, participants suggested several improvements to the app: enabling image enlargement; integrating a geolocation feature for exercise equipment, including demonstrations of both correct and incorrect exercise techniques; and adding an exercise reminder function. Regarding the outdoor practice component, participants suggested that incorporating a supervised outdoor practical session would be beneficial to support their exercise adherence and technique.

**Table 2:** Perceived usefulness of the app (N=13).

Usefulness		Strongly disagree, n (%)	Disagree, n (%)	Neutral, n (%)	Agree, n (%)	Strongly agree, n (%)
**Part 1: User’s exercise performance**						
	Enhanced my knowledge of outdoor exercise facilities.	0 (0)	0 (0)	0 (0)	6 (46)	7 (54)
	Allowed me to execute exercise movements accurately when using outdoor exercise facilities.	0 (0)	0 (0)	0 (0)	4 (31)	9 (69)
	Allowed me to execute exercise movements safely when using outdoor exercise facilities.	0 (0)	0 (0)	0 (0)	7 (54)	6 (46)
	Enhanced my exercise efficiency, enabling me to effectively achieve health benefits, such as improved balance, muscle strength, and overall subjective well-being.	0 (0)	0 (0)	0 (0)	7 (54)	6 (46)
	Assisted me in reaching my physical activity goals.	0 (0)	0 (0)	0 (0)	8 (62)	5 (38)
**Part 2: User’s intention to use the app to support outdoor workouts**						
	When I use the outdoor exercise facilities, I intend to incorporate the Outdoor Rehab-Fit App into my physical activity routine.	0 (0)	0 (0)	0 (0)	6 (46)	6 (46)
	I like to use the Outdoor Rehab-Fit App.	0 (0)	0 (0)	0 (0)	8 (62)	5 (38)
	I will use the Outdoor Rehab-Fit App both during my outdoor workouts and whenever I want to learn more about the outdoor exercise facilities.	0 (0)	1 (8)	1 (8)	7 (54)	4 (31)

**Table 3:** Perceived usefulness of the app content (N=13).

Perceived usefulness		Extremely useless, n (%)	Useless, n (%)	Neutral, n (%)	Useful, n (%)	Extremely useful, n (%)
**Part 3: Mobile app content**						
	Introduction by an exercise specialist	0 (0)	0 (0)	0 (0)	8 (62)	5 (38)
	Tips from a physiotherapist	0 (0)	0 (0)	0 (0)	5 (38)	8 (62)
	Tips from an occupational therapist	0 (0)	0 (0)	0 (0)	5 (38)	8 (62)
	Tips for a caregiver	0 (0)	1 (8)	2 (15)	7 (54)	3 (23)
	Audio guide	0 (0)	0 (0)	2 (15)	7 (54)	4 (31)
	Demonstration video	0 (0)	0 (0)	0 (0)	8 (62)	4 (31)
	Detailed instructions	0 (0)	1 (8)	0 (0)	9 (69)	2 (15)
	Educational video	0 (0)	0 (0)	0 (0)	5 (38)	7 (54)
	Safety precautions and risk management	0 (0)	0 (0)	0 (0)	6 (46)	6 (46)
	Physical fitness examination questionnaire	0 (0)	1 (8)	0 (0)	8 (62)	3 (23)
	Facility location	0 (0)	1 (8)	4 (31)	7 (54)	1 (8)
	My favorites	0 (0)	0 (0)	0 (0)	6 (46)	7 (54)
	The “By Categories” function	0 (0)	0 (0)	0 (0)	5 (38)	7 (54)

**Table 4:** Acceptability of the intervention.

Themes and subthemes					Illustrative quotes		
**The overall perception of the intervention**							
	Appreciate the structure of the intervention, which consists of an app, workshops, and follow-ups				“You need to have all three components, and [each component] complements each other. Once you’re familiar with the app, you can go out and play [with the exercise equipment], right? But I think you still need to attend workshops. Workshops could explain things in more detail. Sometimes, if the app explains things too thoroughly, you might not want to read it.” [P37] “The organization [of the intervention] is compact. It is good. We can ask questions on-site. The physiotherapist and the instructor can show us the exercise movements, which help to correct our previous misconceptions and serve as good encouragement. This gives us the confidence to exercise whenever we have free time or at a scheduled time.” [P29]		
	Increased motivation and confidence in using the free-of-charge OEFs to self-manage physical health				“I really have a lot of issues all over my body, various aches and pains. Although there may be no way to improve them further, I’m still willing to try. Like before, as they said, I’d walk in the park, and if I had time, I’d do a couple of things. But after looking at the app at home, I realized that by taking steps, you can learn the correct way to improve. I really recommend this [the intervention], especially the medical and risk management aspects. For me, it’s really good. From my personal experience, my legs used to be painful when I pedaled the cycling machine, but now, by positioning my toes correctly and not using excessive force, my legs hurt less. My shoulder, too—I don’t know if it was a frozen shoulder or what, but after a year of occupational therapy, it was the same. Using the correct stretching technique, my upper arms feel much more relaxed now. This is one of the things that truly met my expectations.” [P25] “Basically [now] I would attempt to try [the outdoor exercise equipment], explore them, and look at their introductions in the app. For example, if my shoulder feels painful, using these two [outdoor exercise equipment] may help me. For the legs, these [outdoor exercise equipment] are useful for the knees and muscle strength. [If] I don’t need those right now, I can skip them and focus on the ones that help me.” [P7] “Ah, because I knew these [outdoor exercise equipment] were available in the parks, but I did not know there was an app that explained them so clearly. Knowing this [information] gives me more confidence when exercising, and I know it helps me, especially with muscle training and cardiovascular functions. So I think it’s okay.” [P37] “Regarding posture and which exercises could help with certain pain issues in your body, you can focus on those [outdoor exercise equipment] that target your specific needs.” [P44]		
	Improved awareness of the health benefits of using outdoor exercise facilities				“Before joining this intervention, I just passed by the government facilities in parks without knowing much about them and thought they were too simple and childish. I wasn’t interested in trying them out. But after learning about this intervention and its background, I realized that these facilities are actually thoughtfully designed for the older adults. I didn’t know about the government’s intentions [before], but after attending talks delivered by professionals, I felt we shouldn’t waste the effort put into installing these facilities. [If we are not using these facilities], these facilities will become rusty. It seems like such a waste. So, I decided to learn more and engage with them. Even though the movements seem simple, they actually provide good support. On another level, I realized I was too proud, thinking these simple movements were easy. [Now I realize] if you use the correct posture and are willing to spend time understanding how the outdoor exercise equipment can improve your capabilities, it’s really beneficial.” [P45] “I think it’s quite good because you can understand more about the [outdoor sport] facilities available near you.” [P6]		
**Barriers**							
	**App component**						
			Size of the videos		“Some videos [size] are quite small and can’t be enlarged, so they’re not very clear. The equipment shown [in the video] is very small. Even...our phones are small, unless you’re using an iPad. If those [videos] could be enlarged, you could see more details.” [P25]		
			Inability to geolocate the exercise equipment		“The biggest problem is that not all types of outdoor exercise equipment [shown in the app] are available everywhere. I mean, the app categorizes things like strength training and aerobic exercises. But if you want to locate the specific outdoor exercise equipment, this app doesn’t have that information. For example, if I want to do strength training, which park has these facilities? Where are the facilities for various types of training? It doesn’t have this information.” [P8]		
			Unavailability of some exercise equipment		“I think the barrier is...I remember once I went to an outdoor area by the seaside, but I can’t remember the exact location. The explanations on the facilities were blurred, and coincidentally, this [outdoor exercise equipment] wasn’t covered in your app.” [P16]		
	**Outdoor practice component**						
			Equipment being faulty		“And those facilities, especially the ones with pull ropes [upper limb stretcher], are often faulty.” [P8]		
			Hygiene concerns		“Because those places are touched by many people, you need to bring disinfectant wipes to clean them first. When I talked to my friends, they said, ‘Are you kidding me? So many people have used them, the hygiene situation [is relatively bad]...’ So you always have to remember to bring disinfectant wipes.” [P3]		
			Storage problem for belongings		“When I visit the [outdoor exercise facilities], I usually keep my backpack on while I am exercising. It might look a bit awkward, but I don’t want to put it on the ground. Not just in parks, but even in other places, I don’t like putting my things on the ground. I don’t know why, but I’d rather keep it on me than placing it down.” [P3]		
			Other users’ behavior		“Those facilities are installed outdoors, so they’re often affected by the weather and the environment. For example, if it’s too sunny, it can have an impact [reducing people’s willingness to use them].” [P7]		
			Weather		“Those facilities are installed outdoors, so they’re often affected by the weather and the environment. For example, if it’s too sunny, it can have an impact [on reducing people’s willingness in using them].” [P7]		
			Social factors		“If a group of friends or someone like that goes to the park, then I’d be more interested in going there to do that activity. But for now, it’s just me alone, so I don’t have as much motivation.” [P7]		
**Facilitators**							
	**App component**						
		App content facilitates revision and consolidation of new knowledge		“And I think the advantage of having it [the app] is that you can check it anytime, anywhere. For example, if the weather is nice today, I can look at it today or tomorrow. If I go to the park to exercise, I can take it out to look at, or I can look at it before going down. So, there’s no time or space limitation. But if you only attend a class, then I have to rely on my own memory, and I might not remember everything.” [P16]			
		Professional content		“I want to use physiotherapy, occupational therapy, or do exercises myself to improve my physical health, so I try to look for more information on these things. For example, I don’t know if your exercise program will help me, but after listening, I might think, ‘Oh, this is useful and can help me’.” [P7]			
	**Workshop component**						
		Delivery mode		“When it’s an in-person class, like when the coach teaches us how to warm up or when the interventionist shows us [correct] postures, I think this face-to-face instruction is really good.” [P37]			
**Suggestions**							
	**App component**						
		Enable image to be enlarged		“For example, can the image be enlarged? I feel like when I’m exercising, everything looks far away and really small.” [P1]			
		Enable locating certain equipment		“For example, if I want to do strength training, it would be great if you could provide information on where to find these outdoor fitness facilities.” [P8] “Actually, I’ve suggested geo-tagging, so you know where the locations are. The reason is quite simple: I initially had a goal, which is also simple. For example, I want to improve certain joints; I’m not someone who doesn’t move at all...” [P39]			
		Incorporate both incorrect and correct exercise demonstration		“It’s like we need to come here [to attend workshop], but in the future, the workshop will not be available. So, having correct and incorrect demonstrations in the video would allow more in-depth understanding [of how to exercise correctly]. Of course, it would be great if we could all sit here together [to learn the correct posture], right? But I might not have that opportunity. Or in the video, you could include some incorrect demonstrations along with the correct ones, which would help reinforce this [learning outcomes].” [P25]			
		Reminders		“Yes, yes, yes. It’s like when someone asks you, ‘Oh, have you been to so-and-so recently?’ It reminds me. Sometimes people’s mindset is quite interesting. There are certain times when people will ask me if I’m doing something, and I have to commit to doing it. I can’t say I have done it when I actually have not done. So, it becomes a way for me to motivate myself to take action.” [P45]			
	**Outdoor practice component**						
		Provide supervised outdoor practical sessions		“I think that [performing] any type of exercise requires attention to your posture, application of force, and the steps involved. I believe it’s important to have proper guidance to truly achieve the effectiveness of the exercise.” [P8] “For example, if you all have time, you could organize a group to go outdoors and do something together. Like, ‘Hey, on June 2nd, we have an outdoor activity from 2 to 3 PM. Is everyone interested?’ That way, we can gather at Victoria Park and do the activities together in person.” [P45]			

### Results of Secondary Outcomes

**Figure 2:**
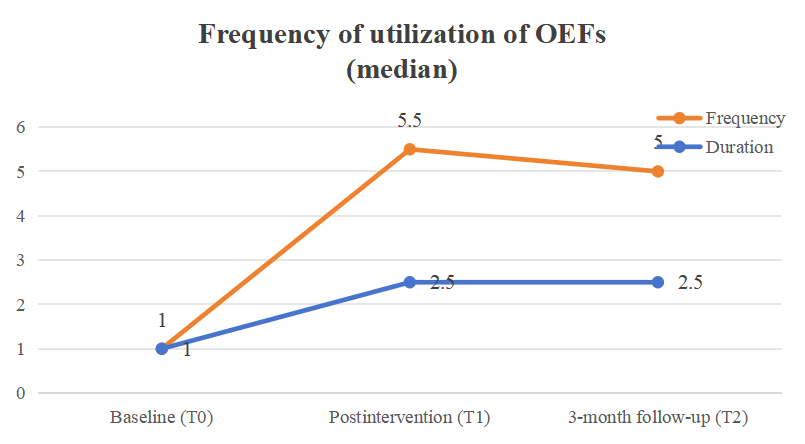
Usage of outdoor exercise facilities (OEFs).

**Figure 3:**
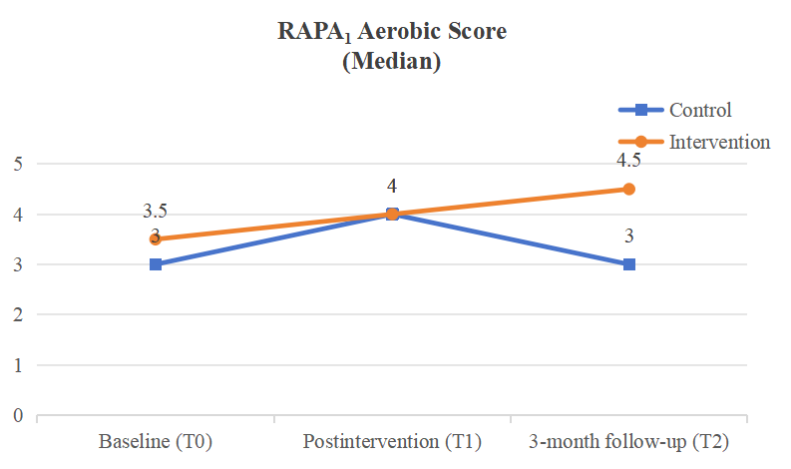
Self-reported physical activity level. RAPA: Rapid Assessment of Physical Activity.

**Figure 4:**
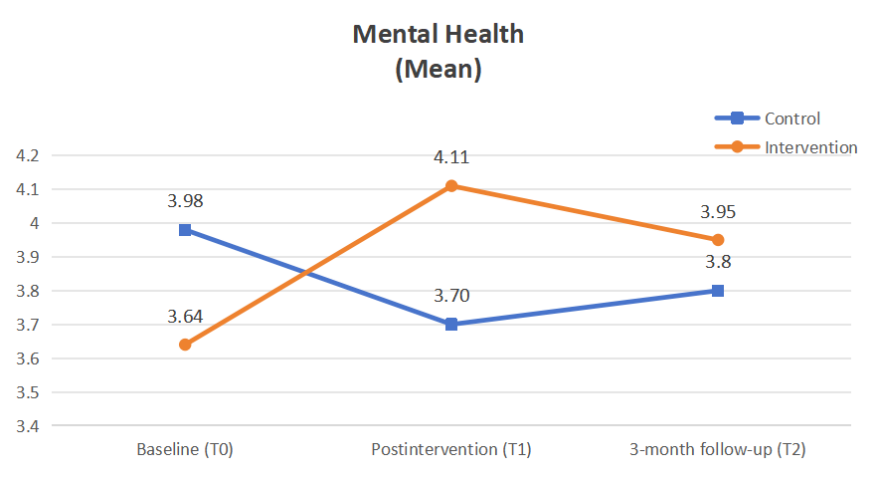
Mental well-being.

Statistically significant improvements from baseline to the 3-month follow-up were observed in 3 of the 5 outcomes in the integrative mHealth intervention group, specifically, usage of OEFs (*P*<0.001), self-reported PA (*P*=0.002), and mental well-being (*P*=0.002; Figures 2-4), while no significant changes were found in any of the 4 outcomes for the active control group. As hypothesized, the intervention group demonstrated a significant increase in OEF usage, reflected in both weekly frequency (*P*<.001, *W*=0.628) and duration of PA (*P*<.001, *W*=0.635), compared with the control group. Additionally, the intervention group showed a significant improvement in self-reported PA level as measured by the RAPA_1_ aerobic score (Wald *χ*^2^_2.96_=21.02, *P*<.001, effect size 1.67). Although objectively measured weekly minutes of MVPA increased from baseline to the 3-month follow-up (147.8, 166.75, and 174.29 minutes per day at T0, T1, and T2, respectively), this change was not statistically significant (*χ*^2^_2.87_=2.29, *P*=.32, effect size 0.15) when compared with the control group. Furthermore, a statistically significant group-by-time effect was observed in favor of the integrative mHealth intervention on mental well-being (Wald *χ*^2^_2.81_=7.24, *P*=.03, effect size 0.29), while changes in exercise self-efficacy from baseline to the 3-month follow-up were not statistically significant for T0, T1, and T2 (5.67, 6.03, and 5.45, respectively; Wald *χ*^2^_2.81_=4.42, *P*=.11, effect size 0.63; Multimedia Appendix 5). Post hoc analyses indicated that the intervention group had significantly higher exercise self-efficacy immediately after the intervention (mean difference 1.42; 95% CI 0.14-2.71; *P*=.03; effect size 0.80) and significantly better self-reported PA levels at the 3-month follow-up (mean difference 1.31; 95% CI 0.52-2.11; *P*<.01; effect size 1.15) compared with the control group (Table 5).

**Table 5:** Effects of the intervention at postintervention (T1) and the 3-month follow-up (T2).

Outcomes measurement			Postintervention (T1)							3-month follow up (T2)				
			Difference (95% CI)		*P* value		Effect size		Difference (95% CI)			*P* value		Effect size
**PA^a^ level**														
	Self-reported PA (RAPA^b^): RAPA_1_ aerobic score	0.21 (–0.65 to 1.06)		.17		0.17		1.31 (0.52 to 2.11)^c^			<.01^c^		1.15^c^	
	Objectively measured PA: MVPA^d^ time (minutes/week)	36.11 (–52.78 to 125.00)		.20		0.30		53.15 (–41.25 to 147.56)			.10		0.41	
**Exercise self-efficacy**														
	SEE-C^e^ scores	1.42 (0.14 to 2.71)^c^		.03^c^		0.80^c^		0.37 (–0.71 to 1.46)			.50		0.25	
**Mental well-being**														
	SWEMWBS^f^ scores	0.27 (–0.29 to 0.82)		.35		0.36		0.07 (–0.37 to 0.52)			.74		0.11	

^a^PA: physical activity.

^b^RAPA: Rapid Assessment of Physical Activity Scale.

^c^Significant in favor of the intervention group.

^d^MVPA: moderate-to-vigorous physical activity.

^e^SEE-C: Exercise Self-Efficacy Scale—Chinese.

^f^SWEMWBS: Short Warwick-Edinburgh Mental Well-Being Scale.

## Discussion

### Principal Findings

This study aimed to assess the feasibility of conducting a full-powered RCT to evaluate the integrative mHealth intervention and to identify any necessary modifications before its implementation. The main findings indicated a high recruitment rate, a marginally acceptable intervention completion rate, and an acceptable attrition rate. Overall, the intervention was well accepted, as reflected in the semistructured interviews. Participants in the intervention group identified barriers and facilitators related to the integrative mHealth intervention and offered suggestions for its improvement. Exploratory analyses of the intervention’s potential effects supported the study hypotheses; however, the feasibility outcomes suggested that the protocol requires modification before conducting a future trial.

### Feasibility of the Intervention

#### Recruitment, Attrition, and Intervention Completion Rates

Our study achieved a recruitment rate of slightly over 50% (38/75, 51%), defined as the percentage of individuals who enrolled in the intervention out of those approached. This rate exceeds those reported in previous lifestyle intervention studies targeting older adults with frailty, which averaged around 20% [47,48], indicating the effectiveness of our recruitment strategies. The attrition rates were 3 out of 19 (16%) in the intervention group and 1 out of 19 (5%) in the control group. Although the attrition rates in both groups were lower than those reported in previous PA interventions for older adults with frailty (>20%) [49,50], the higher attrition rate in our intervention group compared with the control group may suggest the presence of “selective attrition.” Notably, 2 participants dropped out immediately after being allocated to the integrative mHealth intervention group, as the researchers lost contact with them following the communication of randomization results. This may indicate that these participants lacked the motivation to engage with mHealth as a means to improve their health behavior [51]. Regarding the intervention completion rate, the overall completion rate—comprising workshop attendance, self-reported app engagement, and self-reported outdoor practice—was 134 out of 170 (78.8%). Specifically, the outdoor practice completion rate (36/51, 71%), app engagement (35/51, 69%), and workshop attendance (63/68, 93%) were well over 60%. When comparing the workshop components, both the intervention and control groups demonstrated similarly high completion rates (active control: 72/76, 95%; integrative mHealth: 63/68, 93%). These high workshop attendance rates in both groups suggest that participating in workshops to gain health knowledge or exercise experience is highly acceptable among community-dwelling older adults. Although direct comparisons of intervention completion rates across different mHealth studies are challenging—due to variations in intervention components (eg, some include only an app, while others add workshops and self-practice sessions) and differing definitions of app engagement [52]—it is noteworthy that the self-reported app engagement rate (35/51, 69%) in our study was lower than the 88.4% app log-in rate reported in a similar mHealth intervention for older adults with frailty in Japan [48]. Although participants perceived the app as useful, as reflected in the self-developed questionnaire responses, the marginally acceptable adherence suggested usability issues. Qualitative feedback highlighted that app usability could be enhanced by improving its visuals and functionality. Before a future trial, an upgraded app version should incorporate features such as image enlargement, exercise reminders, assistance with locating specific OEFs, and detailed guidance on correct and incorrect exercise postures. These enhancements are expected to improve user engagement and the overall usability of the app. To increase app engagement among older adults with prefrailty and frailty, the overall user experience should be optimized before implementing a full-powered trial. Regarding the outdoor practice component, the completion rate of self-reported outdoor exercise (134/170, 78.8%) was comparable to the logged workout rate (72%) reported in a previous mHealth intervention involving outdoor gym workouts for inactive adults [16]. Qualitative data from semistructured interviews suggest that incorporating at least one supervised outdoor practical session in future interventions could enhance the overall participant experience. To improve adherence to the outdoor practical sessions, future trials should identify specific outdoor gyms located near the community centers and offer supervised outdoor practical sessions alongside the educational workshops held within these centers.

#### Safety

The adverse events reported in our study included a slipping incident caused by faulty outdoor exercise equipment and musculoskeletal pain experienced after using certain exercise devices. The participant who reported musculoskeletal pain had a history of occasional, nonspecific low back and knee pain before participating in the intervention. After advising the participant to reduce the range of motion and exercise at a slower tempo to maintain proper posture and body alignment, the pain resolved. Previous studies on exercise apps [53] and exercise interventions [54] have reported musculoskeletal pain as a common issue among older adult participants. Additionally, research indicates that 66.5% of OEF users experience injuries related to faulty equipment or improper usage [55]. These findings offer valuable insights for improving safety in future full-scale trials. Interventionists with expertise in exercise and health should advise participants with frailty to begin exercising with appropriate dosage, type, and intensity. The principle of gradual exercise progression should be emphasized to help prevent musculoskeletal injuries. Participants should also be reminded to inspect exercise equipment before each use.

#### Acceptability

The current integrative mHealth intervention demonstrated a level of acceptability comparable to that reported in a previous mHealth intervention for older adults with frailty [48] and an OEF-based mHealth intervention for inactive adults [16]. Consistent with the findings of Jansson and colleagues [16], our participants perceived the app content as sufficient to support and motivate unsupervised PA in park settings. Additionally, participants appreciated the intervention’s comprehensive structure, which included 3 components: the app, workshops, and unsupervised outdoor practice. This finding aligns with recent research indicating that interventions combining workshop and digital components yield more promising outcomes in older adults than those relying on a single component [56,57]. While the app was generally found to be acceptable—reflected in responses to the self-developed questionnaire and semistructured interviews—the results also provided important insights for further improving both the app and the overall integrative mHealth intervention before future trials. Unlike the study by Jansson and colleagues [16], which suggested that the Ecofit app could effectively support unsupervised PA in park settings, participants in our study—who were community-dwelling older adults with prefrailty and frailty—recommended incorporating at least one supervised session in parks equipped with OEFs.

### Preliminary Effects of the Intervention

#### Overview

This study explored the preliminary effects of an integrative mHealth intervention on older adults with prefrailty and frailty, focusing on the use of OEFs, PA levels, exercise self-efficacy, and mental well-being. Given the exploratory and underpowered nature of the analysis, results should be interpreted with caution. To provide insights into the magnitude and direction of effects, results are presented alongside 95% CIs [58,59].

#### Self-Reported and Objectively Measured PA Levels

Sustaining PA behavioral change among older adults with prefrailty and frailty is challenging, as demonstrated in previous research [12]. Therefore, the sustained increase in PA levels—measured both subjectively and objectively—at the 3-month follow-up in our study is an encouraging finding. This result aligns with a prior outdoor gym mHealth study, which showed that a mobile app providing education on OEFs effectively maintained resistance training–specific PA behavior changes among inactive adults at 3 and 9 months [21]. While the integrative mHealth intervention demonstrated potential treatment effects, it is important to note that only a subsample of participants agreed to wear accelerometers to objectively measure PA. At baseline, 16 out of 18 (89%) in the control group and 15 out of 16 (94%) in the intervention group consented to wear the device, but only 24 out of 34 (71%) of these participants wore it across all time points. This level of compliance is consistent with previous PA studies involving older adults [60]. To enhance accelerometer adherence in future trials, providing financial incentives and ensuring logistical convenience will be essential.

#### Mental Well-Being and Exercise Self-Efficacy

The observed improvements in mental well-being at the 3-month follow-up and exercise self-efficacy immediately postintervention align with findings from previous OEF-based PA and mHealth studies [14,61]. However, these results require confirmation in a larger trial. Notably, 5 (15%) participants showed signs of cognitive impairment that prevented them from completing assessments related to mental well-being and exercise self-efficacy. Therefore, future trials should consider excluding individuals with cognitive impairment to ensure data accuracy.

### Novelty of the Integrative mHealth Intervention of This Study

Although integrative health interventions supporting the use of public OEFs to promote PA are not new, this study presents the first integrative mHealth intervention specifically designed for community-dwelling older adults with prefrail or frail health status. The intervention aimed to improve PA, exercise self-efficacy, and mental well-being in this population through the combined use of an app, educational workshops, and unsupervised outdoor engagement with senior-friendly OEFs available in public open spaces. The novelty of this integrative mHealth intervention lies in its unique combination of PA and rehabilitation knowledge. The “Outdoor Rehab-Fit” app is the first to offer rehabilitation insights and guidance from physiotherapists and occupational therapists for each piece of outdoor exercise equipment available in public open spaces in Hong Kong. This added rehabilitation perspective helps older adults better understand how using specific outdoor equipment can guide movements and activities that improve their physical condition. For example, using the “calf stretch” equipment (an inclined ramp) can help alleviate foot and knee joint pain by improving calf flexibility, while exercising with the “bench stepper” equipment can strengthen the quadriceps and gluteus maximus muscles, ultimately enhancing walking and stair-climbing abilities. Unlike previous apps that primarily focus on fitness enhancement [21], the current app aims to boost users’ expectations regarding how OEFs can address functional limitations while also increasing PA levels. This intervention acknowledges that health concerns and functional limitations are significant barriers to exercise among this population [62].

Additionally, the integrative delivery structure is another novel aspect of the current intervention. Unlike previous outdoor gym mHealth interventions designed for adults, which included only a single introductory workshop [21], this intervention emphasizes the importance of a comprehensive approach by incorporating 4 educational workshop sessions alongside the app. These workshops recognize that older adults generally have lower digital literacy compared with the broader adult population, thereby providing more support to facilitate effective app use and engagement. Older adults require real-time, interactive support not only to learn how to use the app but also to gain knowledge relevant to their engagement with OEFs. The workshop sessions complement the app by delivering educational content and offering opportunities for real-time interaction, enabling older adults with frailty to better understand essential exercise concepts tailored to their needs. The high adherence to the workshops observed in this study underscores the importance of this integrated delivery approach.

### Implications to Future Large-Scale Study

The pilot trial provides preliminary evidence that the proposed integrative mHealth intervention may promote sustainable improvements in PA, mental well-being, and exercise self-efficacy among older adults with prefrailty and frailty. The demonstrated effectiveness, along with the widespread availability of OEFs in the region, supports the scalability of this intervention. However, the feasibility and acceptability findings from this study highlight areas of the intervention that require refinement before conducting a future full-scale trial. Future large-scale trials should be conducted in community settings in collaboration with local community organizations and utilize a cluster RCT design. To improve adherence to the outdoor practice component, the intervention should incorporate at least one supervised outdoor session at a nearby outdoor gym, in addition to educational workshops held at the community center. Enhancing app adherence will require usability improvements, including providing exercise demonstrations in video format with the option to enlarge for better visibility. Additionally, the app should incorporate a reminder function to prompt users about their exercise routines. The educational content within the app also requires updating to enhance its relevance and usefulness. To improve safety, future interventions should include guidance and reminders for participants on how to inspect exercise equipment before each use. Workshops should emphasize the concept of gradual exercise progression tailored to each piece of equipment. Regarding PA measurement, our study observed a decline in accelerometer compliance over repeated time points. Therefore, future trials should consider using self-reported PA measurement tools with strong psychometric properties to ensure higher compliance in longitudinal assessments. Lastly, because the study was conducted within a university setting, it naturally attracted participants with higher education levels and greater digital literacy. Future community-based trials are likely to recruit individuals with lower digital literacy. To support these participants in the integrative mHealth group, it will be essential to mobilize adequate social assistance from community volunteers or university students [63].

### Limitations

This study has several limitations. First, the integrative mHealth intervention consisted of 3 components: the mobile app, OEFs, and workshops. Consequently, isolating the effects of each component was challenging. However, the inclusion of an active control group, which matched contact hours and exercise knowledge, helped minimize confounding factors and allowed for a clearer estimation of the intervention’s potential treatment effect. Second, the study required participants to own a smartphone, excluding those without one. However, smartphone penetration in Hong Kong is high, with 97.1% of the population aged 10 years and above owning a smartphone [64]. Additionally, the mandatory use of the Leave Home Safe app during the 2020 COVID-19 pandemic further increased smartphone ownership among older adults [65]. Third, participants had access to varying numbers and types of outdoor exercise facilities (OEFs) in their neighborhoods, which may have influenced their engagement with the intervention. It is impossible to standardize the built physical environment, and this variation among neighborhoods may have influenced the study outcomes. Nevertheless, this pilot feasibility RCT estimated potential treatment effects within a real-world context. Finally, it is important to emphasize that the results of this pilot RCT provide only preliminary evidence regarding the feasibility of the integrative mHealth intervention for community-dwelling older adults with prefrailty and frailty.

### Strengths

To the best of our knowledge, this is the first study to examine the feasibility of an integrative mHealth intervention combining an app, workshops, and unsupervised outdoor practice specifically for community-dwelling older adults with prefrailty and frailty. The study gathered valuable feasibility and acceptability data to inform important modifications ahead of a full-powered trial. Additionally, a health education workshop with exercise experiential sessions—commonly offered in community centers for older adults—served as the comparator group, allowing for a meaningful comparison. Finally, the study protocol was registered before data collection, ensuring transparency in all aspects of reporting.

### Conclusion

Preliminary evidence supports the foundation of this study; however, strategies to improve intervention adherence are needed, and modifications should be made before future trials.

## Appendix

Multimedia Appendix 1 CONSORT eHEALTH checklist (V 1.6.1).

Multimedia Appendix 2 Intervention completion assessment.

Multimedia Appendix 3 Self-developed questionnaire on the perceived usefulness of the mobile app.

Multimedia Appendix 4 Interview guide.

Multimedia Appendix 5 Secondary outcomes of both the intervention and control groups at baseline, postintervention, and the 3-month follow-up.

## Abbreviations

CHAMPS: Community Healthy Activities Model Program for Seniors Physical Activity Questionnaire
CONSORT: Consolidated Standards of Reporting Trials
FRAIL: Fatigue, Resistance, Ambulation, Illness, and Loss of Weight
mHealth: mobile health
MVPA: moderate-to-vigorous physical activity
OEF: outdoor exercise facility
PA: physical activity
RAPA: Rapid Assessment of Physical Activity
RCT: randomized controlled trial
SWEMWBS: Short Warwick-Edinburgh Mental Well-Being Scale

## Data Availability

The data that support the findings of this study are available upon reasonable request from the authors.
