# Supplementary material for: Outdoor Exercise Facility–Based Integrative Mobile Health Intervention to Support Physical Activity, Mental Well-Being, and Exercise Self-Efficacy Among Older Adults With Prefrailty and Frailty in Hong Kong: Pilot Feasibility Randomized Controlled Trial Study
Source: JMIR Mhealth Uhealth. 2025 Jun 5;13:e69259. doi: 10.2196/69259 (PMC12179572; doi:10.2196/69259)
Supplement: Multimedia Appendix 2 [file mhealth_v13i1e69259_app2.pdf]

Supplementary Material 2 Intervention Completion Assessment

Intervention completion record sheet

Note: Fill in "√" or "×" in the form. Fill in "√" to indicate that the intervention has been completed. Otherwise, fill in "x" to indicate that the intervention has not been completed.

ID	Week 1	Week 2			Week 3			Week 4			Completion rate
	Workshop attendance	Workshop attendance	Self-practice	App-self learning	Workshop attendance	Self-practice	App-self learning	Workshop attendance	Self-practice	App-self learning	
