# Supplementary material for: Outdoor Exercise Facility–Based Integrative Mobile Health Intervention to Support Physical Activity, Mental Well-Being, and Exercise Self-Efficacy Among Older Adults With Prefrailty and Frailty in Hong Kong: Pilot Feasibility Randomized Controlled Trial Study
Source: JMIR Mhealth Uhealth. 2025 Jun 5;13:e69259. doi: 10.2196/69259 (PMC12179572; doi:10.2196/69259)
Supplement: Multimedia Appendix 3 [file mhealth_v13i1e69259_app3.pdf]

Supplementary Material 3. Self-developed questionnaire on the perceived usefulness of the mobile app

Perceived Usefulness of Outdoor Rehab-Fit App

Part 1-User's performance

Measurement Items	Select the box that applies to you.				
	Strongly disagree	Disagree	Neutral	Agree	Strongly agree
1. Using Outdoor Rehab -Fit App can enhance my knowledge of outdoor exercise facilities.					
2. Using Outdoor Rehab -Fit App allows me to execute exercise movements accurately when utilizing outdoor exercise facilities.					
3. Using Outdoor Rehab -Fit App allows me to execute exercise movements safely when utilizing outdoor exercise facilities.					
4. Using Outdoor Rehab -Fit App enhances my exercise efficiency (enabling me to effectively achieve health benefits such as improving balance, muscle strength, and overall subjective well-being).					
5. Using the Outdoor Rehab-Fit App can assist me in reaching my physical activity goals.					

Part 2-User's behavior

Measurement Items	Select the box that applies to you.				
	Strongly disagree	Disagree	Neutral	Agree	Strongly agree
1. When I utilize the outdoor exercise facilities, I intend to incorporate the Outdoor Rehab-Fit App into my physical activity routine.					
2. I like to use the Outdoor Rehab-Fit App.					
3. I will utilize the "Outdoor Rehab-Fit App" both during my outdoor workouts and whenever I want to learn more about the outdoor exercise facilities.					

Part 3- Mobile app content

<table>
<tr><th colspan="3" rowspan="2">App Items</th><th colspan="5">Select the response that applies to you.</th></tr>
<tr><th>Extremely useless</th><th>useless</th><th>Neutral</th><th>useful</th><th>Extremely useful</th></tr>
<tr><td rowspan="8">Understanding of the facilities</td><td rowspan="4">Introduction to facilities</td><td>Introduction by Exercise Specialist</td><td></td><td></td><td></td><td></td><td></td></tr>
<tr><td>Tips from Physiotherapist</td><td></td><td></td><td></td><td></td><td></td></tr>
<tr><td>Tips from Occupational Therapist</td><td></td><td></td><td></td><td></td><td></td></tr>
<tr><td>Tips for caregiver</td><td></td><td></td><td></td><td></td><td></td></tr>
<tr><td rowspan="3">Outdoor practice</td><td>Audio guide</td><td></td><td></td><td></td><td></td><td></td></tr>
<tr><td>Demonstration video</td><td></td><td></td><td></td><td></td><td></td></tr>
<tr><td>Detailed Instructions</td><td></td><td></td><td></td><td></td><td></td></tr>
<tr><td>Key highlights</td><td>Educational video</td><td></td><td></td><td></td><td></td><td></td></tr>
<tr><td rowspan="2">Risk management</td><td colspan="2">Reminder of safety precautions and risk management</td><td></td><td></td><td></td><td></td><td></td></tr>
<tr><td colspan="2">Physical fitness examination questionnaire</td><td></td><td></td><td></td><td></td><td></td></tr>
<tr><td rowspan="2">Others</td><td colspan="2">Facility location</td><td></td><td></td><td></td><td></td><td></td></tr>
<tr><td colspan="2">My favorites</td><td></td><td></td><td></td><td></td><td></td></tr>
</table>
