# Supplementary material for: Outdoor Exercise Facility–Based Integrative Mobile Health Intervention to Support Physical Activity, Mental Well-Being, and Exercise Self-Efficacy Among Older Adults With Prefrailty and Frailty in Hong Kong: Pilot Feasibility Randomized Controlled Trial Study
Source: JMIR Mhealth Uhealth. 2025 Jun 5;13:e69259. doi: 10.2196/69259 (PMC12179572; doi:10.2196/69259)
Supplement: Multimedia Appendix 4 [file mhealth_v13i1e69259_app4.pdf]

Supplementary file 4. Interview guide

Integrative mHealth Intervention

1. What expectations did you have before joining the program?
   [Prompt：1. Know more about outdoor exercise facilities 2. Increase physical activity level]
2. Please describe your overall experience with the program.
3. How successful do you think the program has been for you?
4. What barriers do you encounter in completing the program?
   [Prompt: 1. Operation of the mobile App 2. Usage of outdoor exercise facilities]
5. What are the facilitators of this program?
   [Prompt: 1. App 2. Face-to-face session]
6. Based on your experience in this program, would you recommend this program to others? And why?

About the mobile App

7. Have you encountered any difficulties when you use the app?
   a. [Prompt: 1. The layout and design are not ideal 2. The information provided is hard to understand]
8. What are your specific goals when using this mobile app? Have these goals been achieved? [Prompt: 1. Increase knowledge of different types of outdoor exercise facilities 2. Exercise with these equipment safely and effectively]

Recommendation

9. Do you have any suggestions for this program as a whole?
10. Do you have any suggestions for this mobile App as a whole?

[Prompt：1. Functionality 2. The aesthetic layout and design 3. The comprehensiveness of information provided 4. The authority and authenticity of the information provided]
