# Supplementary material for: Outdoor Exercise Facility–Based Integrative Mobile Health Intervention to Support Physical Activity, Mental Well-Being, and Exercise Self-Efficacy Among Older Adults With Prefrailty and Frailty in Hong Kong: Pilot Feasibility Randomized Controlled Trial Study
Source: JMIR Mhealth Uhealth. 2025 Jun 5;13:e69259. doi: 10.2196/69259 (PMC12179572; doi:10.2196/69259)
Supplement: Multimedia Appendix 5 [file mhealth_v13i1e69259_app5.docx]

| Outcomes | N | Mdn (IQR)/Mean±SE | | | | | | Baseline comparison | | | | Time effect | | | | Group-by-time effect | | | | Effect size | |
| --- | --- | --- | --- | --- | --- | --- | --- | --- | --- | --- | --- | --- | --- | --- | --- | --- | --- | --- | --- | --- | --- |
|  |  | Baseline (T0) | Postintervention (T1) | Mean difference (95% CI) (T0-T1) | 3-month follow-up (T2) | Mean difference (95% CI) (T0-T2) | t/z | | P-value | | Wald χ^2^ | | P-value | | Wald χ^2^ | | P-value | | Cohen’s d | |  |
| **Physical activity level** | | | | | | | | | | | | | | | | | | | | | |
| Utilization of OEFs (Mdn, IQR) | | | | | | | | | | | | | | | | | | | | | |
| Frequency | 16 | 1(2) | 5.5(2) |  | 5(2) |  | 20.11 | | < .001 | |  | |  | | - | | - | | .63 | |  |
| Duration | 16 | 1(1) | 2.5(2) |  | 2.5(2) |  | 20.32 | | < .001 | |  | |  | | - | | - | | .64 | |  |
| Self-reported physical activity by RAPA | | | | | | | | | | | | | | | | | | | | | |
| RAPA_1_ aerobic score (Mdn, IQR) | | | | | | | | 1.365^a^ | | .21 | | 5.84 | | .054 | | 21.02 | | < .001 | | 1.67 | |
| Control group | 18 | 3(0.5) | 4(2) | .54 [-.27,1.34] | 3(0.5) | -.09 [-.78,.59] |  | |  | |  | |  | |  | |  | |  | |  |
| Intervention group | 16 | 3.5(1) | 4(0) | .47 [-.02,0.96] | 4.5(1) | .93 [.39,1.47] |  | |  | |  | |  | |  | |  | |  | |  |
| Physical activity objectively measured using an ActiGraph | | | | | | | | | | | | | | | | | | | | | |
| MVPA [Mean±SE] | | | | | | | | | | | | | | | | | | | | | |
| MVPA (min/week) | | | | | | | | 0.712^a^ | | .50 | | 1.48 | | .48 | | 2.29 | | .32 | | .15 | |
| Control group | 16 | 131.07±30.63 | 130.64±31.12 | -.43 [-23.67,22.8] | 121.14±35.00 | -9.93 [-51.76,31.89] |  | |  | |  | |  | |  | |  | |  | |  |
| Intervention group | 15 | 147.8±27.04 | 166.75±30.21 | 18.95 [-.08,37.97] | 174.29±29.63 | 26.49 [-13.01,65.99] |  | |  | |  | |  | |  | |  | |  | |  |
| **Exercise self-efficacy [Mean ± SE]** | | | | | | | | 0.928^b^ | | .36 | | .16 | | .92 | | 4.42 | | .11 | | .63 | |
| Control group | 14 | 4.94±0.68 | 4.93±0.52 | -.02 [-1.63,1.59] | 5.40±0.42 | .45 [-.49,1.39] |  | |  | |  | |  | |  | |  | |  | |  |
| Intervention group | 15 | 5.67±0.42 | 6.03±0.36 | .36 [-.53,1.24] | 5.45±0.31 | -.22 [-.92,.48] |  | |  | |  | |  | |  | |  | |  | |  |
| **Mental well-being [Mean ± SE]** | | | | | | | | 1.578^b^ | | .13 | | .81 | | .67 | | 7.24 | | .03 | | .29 | |
| Control group | 14 | 3.98±0.11 | 3.78±0.27 | -.20 [-.64,.23] | 3.82±0.21 | -.16 [-.49,.16] |  | |  | |  | |  | |  | |  | |  | |  |
| Intervention group | 15 | 3.68±0.16 | 4.11±0.17 | .44 [.15,.73] | 3.96±0.13 | .29 [.09,.49] |  | |  | |  | |  | |  | |  | |  | |  |
| Note: Mdn, median; IQR, interquartile range; SE, standard error; OEFs: outdoor exercise facilities; RAPA, The Rapid Assessment of Physical Activity Scale; MVPA, Moderate-to-vigorous physical activity. ^b^Independent samples t-test. The results are reported by adjusting for gender and falls in the past 6 months. Effect size: Cohen’s d | | | | | | | | | | | | | | | | | | | | | |
